# Doctor Claims He Cures Tuberculosis

**Published:** 1914-11

**Authors:** 


### Doctor Claims He Cures Tuberculosis.

BATTLE CREEK, MICH., September 16, 1914.

After curing between 750 and 1000 pulmonary tuberculosis cases, his own included, Dr. J. D. Gibson, of Denver, announced today before the twenty-fourth annual convention of the American Electro Therapeutic Association that after thirteen years' experimentation he considered the great white plague curable, and prophesied it would be wiped out in ten years.

Dr. George E. Pfahler, of Philadelphia, President of the organization, took issue with Dr. Frederic C. Tice, Roanoke, Va., who declares he has cured thirty cases of typhoid with X-ray and light. The controversy became so warm that Dr. Tice challenged any four physicians in the United States to bring him a case of typhoid in which he cannot reduce the fever in forty-eight hours and have the patient on his feet in ten days. He put up $100 and demands the four to do the same.—*Austin, American,* September 17, 1914.

